# Tailoring Gellan Gum Spongy-Like Hydrogels’ Microstructure by Controlling Freezing Parameters

**DOI:** 10.3390/polym12020329

**Published:** 2020-02-05

**Authors:** Helena R. Moreira, Lucília P. da Silva, Rui L. Reis, Alexandra P. Marques

**Affiliations:** 1 3B’s Research Group, I3Bs–Research Institute on Biomaterials, Biodegradables and Biomimetics, University of Minho, Headquarters of the European Institute of Excellence on Tissue Engineering and Regenerative Medicine, 4805-017 Guimarães, Portugal; helena.moreira@i3bs.uminho.pt (H.R.M.); lucilia.silva@i3bs.uminho.pt (L.P.d.S.); rgreis@i3bs.uminho.pt (R.L.R.); 2 ICVS/3B’s–PT Government Associate Laboratory, 4805-017 Braga/Guimarães, Portugal; 3 The Discoveries Centre for Regenerative and Precision Medicine, Headquarters at University of Minho, Avepark, Barco, 4805-017 Guimarães, Portugal

**Keywords:** spongy-like hydrogels, freezing device, microstructure

## Abstract

Gellan gum (GG) spongy-like hydrogels have been explored for different tissue engineering (TE) applications owing to their highly attractive hydrogel-like features, and improved mechanical resilience and cell performance. Although the whole process for the preparation of these materials is well-defined, we hypothesized that variations occurring during the freezing step lead to batch-to-batch discrepancies. Aiming to address this issue, two freezing devices were tested, to prepare GG spongy-like hydrogels in a more reproducible way. The cooling and freezing rates, the nucleation time and temperature, and the end freezing time were determined at different freezing temperatures (−20, −80, and −210 °C). The efficacy of the devices was assessed by analyzing the physicochemical, mechanical, and biological properties of different formulations. The cooling rate and freezing rate varied between 0.1 and 128 °C/min, depending on the temperature used and the device. The properties of spongy-like hydrogels prepared with the tested devices showed lower standard deviation in comparison to those prepared with the standard process, due to the slower freezing rate of the hydrogels. However, with this method, mean pore size was significantly lower than that with the standard method. Cell entrapment, adhesion, and viability were not affected as demonstrated with human dermal fibroblasts. This work confirmed that batch-to-batch variations are mostly due to the freezing step and that the tested devices allow fine tuning of the scaffolds’ structure and properties.

## 1. Introduction

Porous biomaterials have been extensively used in tissue engineering, holding a great promise for the regeneration and repair of damaged tissues as providers of a three-dimensional structure (3D) for the adhesion, growth, migration, and proliferation of cells [1]. Higher pore size and porosity is generally associated with higher cellular performance [2,3], but attaining the right balance between the pore size and overall porosity still is critical [4]. The architecture and isotropy of scaffolds also influence overall cell behavior, as cells randomly distribute and project filopodia in scaffolds with random pores but elongate along the pores in radially or axially aligned scaffolds [5]. Consequently, host response and neotissue formation have also been found to be influenced by scaffolds’ microstructure [6,7,8], particularly the size [9] and direction [10] of the pores. Although endothelial cells enter and new vessels can be formed through 30–40 μm pores [11,12], higher pore sizes are needed for the full colonization of the structure and efficient diffusion of oxygen, nutrients, and metabolites to the cells to nourish them prior the establishment of a vascular network [13,14]. Higher porosity is also associated with a higher area to support cell adhesion in the first stage, and with faster biodegradability, hence providing additional space for tissue ingrowth [9]. Moreover, scaffolds with multidirectional pores endorse the deposition of non-fibrotic loose and randomly organized collagen fibers in contrast to unidirectional scaffolds [10]. Therefore, small alterations in the scaffolds’ microarchitecture impact cell–material interactions and subsequent tissue formation. Hence, a precise control of the pore size, architecture, and interconnectivity of scaffolds is needed.

Gellan gum (GG) spongy-like hydrogels have been previously proposed by us; these structures are characterized by a water content comparable to hydrogels, but improved resilience to deformation and mechanical stability, as well as unprecedented cell-adhesive features [15]. These materials have already demonstrated impressive results regarding the adhesion of cells from different origins, such as skin [16], bone [17], and muscle [18,19]. A proven impact was also shown in terms of in vivo skin repair, where a positive effect over the re-epithelization [20,21] and neovascularization [22,23] of full-thickness wounds was observed. Spongy-like hydrogels are prepared from precursor hydrogels through a well-defined processing methodology that includes freezing and freeze-drying the hydrogels to give rise to dried polymeric networks. During freezing, the water already existing in the hydrogels forms ice crystals that are sublimated during freeze-drying, thus creating a porous dried polymeric structure with a particular microarchitecture [24]. These are able to rapidly uptake any solution giving rise to spongy-like hydrogels that comprise features between sponges and hydrogels. While the whole procedure for the spongy-like hydrogels’ formation is well-defined, there are batch-to-batch discrepancies that we hypothesized to result from the temperature variations occurring during the freezing stage. In fact, different parameters such as the cooling rate, the final freezing temperature, and the thermal hold were shown to affect the scaffolds’ properties, particularly the pore size [25,26,27,28,29].

Therefore, in this work we tested the effect of two freezing devices, in relation to the standard method, over the pore size of GG dried polymeric networks and spongy-like hydrogels prepared at –20, –80, and –210 °C. The changes of the freezing profiles were analyzed in order to understand the contribution of each freezing parameter. Moreover, different concentrations of GG were used to eliminate potential effects related to the amount of polymer. The maintenance of the characteristic features of spongy-like hydrogels such as cell-adhesive nature was further confirmed.

## 2. Materials and Methods

### 2.1. Freezing Device

**Figure 1:**
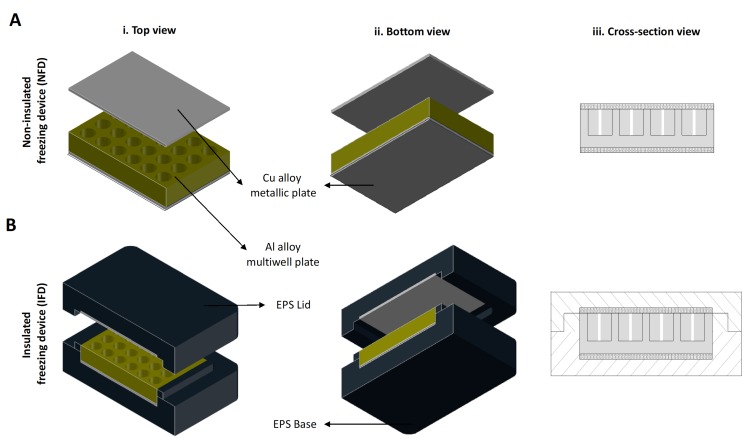
Scheme of the (i) top, (ii) bottom and (iii) cross-section view of the freezing devices. (**A**) The non-insulated freezing device (NFD) contains a conductive aluminum multiwell plate, with a cover and a base of a thermo-conductive copper alloy. (**B**) The insulated freezing device (IFD) is composed by the same components of the NFD and an insulating outer shell. EPS—expanded polystyrene.

The insulated freezing device (IFD) contains a conductive aluminum multiwell plate, with a cover and a base of a thermo-conductive copper alloy and of an insulator. The non-insulated freezing device (NFD) is composed of the same components as the IFD except the insulating outer shell (Figure 1). The conductive multiwell plate (aluminum 6082-T6 alloy, Al) radiates the heat within the plate maintaining an even temperature in all wells. Directly under and above the multiwell plate is placed a layer of a C11000 copper alloy (Cu), a thermo-conductive material to allow a faster and more uniform transfer of thermal energy. The outer shell is constructed from expanded polystyrene (EPS), a thermal insulating material, preventing/reducing vapor formation from an external surface of the device and reducing heat transfer due to the operator’s physical contact. A tissue culture polystyrene (TCPS) multiwell plate without any other cover was used in the standard method as previously reported by us [15].

### 2.2. Spongy-Like Hydrogel Preparation

GG spongy-like hydrogels were prepared as previously described [15]. Briefly, Gelzan powder (Sigma-Aldrich, Saint Louis, MI, USA) was dissolved in deionized water (0.75% and 1.25% (*w*/*v*)), under stirring at 90 °C. After dissolution, the solution was cast into the desired molds (TCPS 24-well plate—standard method, and aluminum 24-well plate—IFD and NFD) and rapidly mixed with the crosslinking solution, cell culture alpha-minimal essential medium (α-MEM, Gibco, Carcavelos, Portugal) with no further supplementation. After the hydrogel formation, they were stabilized in phosphate buffered saline (PBS, Sigma-Aldrich, Algés, Portugal) solution for 48 h. Afterward, hydrogels were frozen at −210 °C in liquid nitrogen (N_2_) for 20 min, or at −20 or −80 °C for 18−20 h before being freeze-dried (LyoAlfa 10/15, Telstar, Terrassa, Spain) for three days to obtain GG dried polymeric structures.

### 2.3. Thermal Profile Analysis

**Figure 2:**
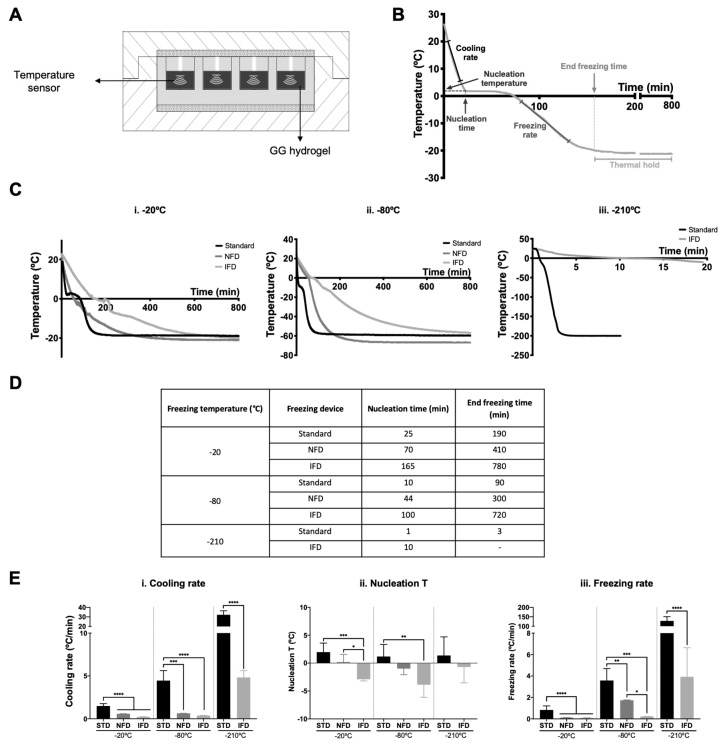
Freezing profiles of 1.25% gellan gum (GG) hydrogels. (**A**) Cross-section of the insulated freezing device with GG hydrogels. The temperature curves were measured using a temperature sensor that was placed inside the hydrogel. (**B**) Schematic of a thermal profile of a GG hydrogel with the cooling, nucleation temperature (T) and time, freezing rate, and end freezing time marked. (**C**) Mean temperature profiles of GG hydrogels during freezing at (i) −20, (ii) −80, and (iii) −210 °C with the three different freezing molds. (**D**) Characteristic values of the freezing of GG hydrogels. (**E**) Effect of both temperature and freezing mold on the (i) cooling rate, (ii) nucleation T, and (iii) freezing rate of GG hydrogels. STD refers to materials prepared with the standard method. * *p* < 0.05, ** *p* < 0.01, *** *p* < 0.001, **** *p* < 0.0001, one-way ANOVA with Bonferroni multiple comparison post-test.

The thermal profile along the freezing of the hydrogel was determined using temperature sensors (Innovative Sensor Technology AG, Ebnat-Kappel, Switzerland) that were positioned in the middle part of seven hydrogels randomly distributed throughout the wells. Measurements were recorded every 5 s using a computer program written in Arduino (v1.8.10, Somerville, MA, USA). The cooling rate, nucleation time and temperature, freezing rate, and end freezing time were determined from the thermal profiles (Figure 2A,B).

### 2.4. Microscopic Analysis

Scanning electron microscopy (SEM) was used to analyze the microstructure of the dried polymeric networks. Prior to analysis, samples were sputter coated with a mixture of gold–palladium. A JSM-6010LV (JEOL, Akishima, Japan) microscope, operating with an accelerating voltage of 15 kV was used to capture images.

Confocal microscopy was used to analyze the microstructure of spongy-like hydrogels (hydrated dried polymeric structures) after staining with 4′,6-diamidino-2-phenylindole (DAPI, 0.2 mg/mL, Biotium, Alfragide, Portugal) for 2 h at room temperature (RT), as previously described by da Silva et al. [30]. Samples were observed with a Leica TCS SP8 confocal microscope (Leica, Mannheim, Germany). FIJI for ImageJ (version 2.0.0-rc-69, NIH, Baltimore, MD, USA) was used to measure the pore size in five random images for each condition (three independent experiments).

### 2.5. Micro-Computed Tomography (μ-CT)

The spongy-like hydrogels’ microarchitecture was analyzed using a high-resolution X-ray microtomography system SkyScan 1072 scanner (SkyScan, Kontich, Belgium). Samples were scanned in high-resolution mode using a pixel size of 11.31 μm (magnification of 23.30×) and an integration time of 1.7 s. The x-ray source was set at 35 keV of energy and 215 μA of current. Representative datasets of 150 slices were transformed into a binary picture using a dynamic threshold of 45e225 (gray values) to distinguish polymer material from pore voids. Pore size was obtained using the CT Analyzer (v1.5.1.5, SkyScan).

### 2.6. Compressive Tests

The mechanical behavior of spongy-like hydrogels (hydrated in PBS for 24 h, at RT) was tested, under static compression using an Instron 5543 (Instron, Norwood, MA, USA). Samples (13.4 mm diameter and 3 mm height) were submitted to a pre-load of 0.1 N and tested up to 60% of strain, at a loading rate of 2 mm/m. The compressive modulus was determined from the most linear part of the stress/strain curves using the secant method.

### 2.7. Water Uptake Quantification

Dried polymeric networks were immersed in PBS or α-MEM up to 24 h at 37 °C, to determine the water uptake profile. Samples were weighed prior immersion (Wd) and after each time point (Ww) to calculate the percentage of water uptake along time (Equation (1)). The water content was determined for the end time point using the same equation.

Water uptake (%) = (Ww − Wd)/Wd × 100(1)

### 2.8. Cell Isolation and Culture

Human skin was obtained from abdominoplasty procedures of healthy donors performed at Hospital da Prelada (Porto) after patient’s informed consent and under a collaboration protocol approved by the ethical committees of both institutions. Skin specimens were cut into small fragments and incubated with dispase (BD Biosciences, Alfragide, Portugal) overnight at 4 °C. The epidermis was peeled off and the dermis was digested for 3 h at 37 °C using collagenase type II (125 U/mL, Sigma-Aldrich, Algés, Portugal). The resulting cell suspension was filtered through a 70 μm cell strainer and centrifuged at 1500 rpm for 10 min at 4 °C and placed in culture. Human dermal fibroblasts (hDFbs) were cultured in α-MEM, supplemented with 10% fetal bovine serum (FBS, Gibco, Carcavelos, Portugal) and 1% antibiotic/antimycotic (Gibco, Carcavelos, Portugal) at 37 °C in a humidified atmosphere with 5% CO_2_. hDFbs were used at passage 3 and 4.

### 2.9. Cell Seeding/Entrapment

The hDFbs cell suspension (5 × 10^5^ cells) was prepared in α-MEM (100 μL). For cell seeding/entrapment, cell suspension was dispensed dropwise on the top of dried polymeric networks. Constructs were incubated for 30 min, at 37 °C, 5% CO_2_ to allow maximum cell entrapment within the structures before fresh medium was added to the well up to a total volume of 1 mL.

### 2.10. DNA Quantification

The cell seeding/entrapment efficiency in the spongy-like hydrogels was determined based on the amount of DNA quantified after 24 h of cell culture (DNA_f_) in relation to the quantity corresponding to the cell seeding density (DNA_i_). The PicoGreen dsDNA assay kit (Invitrogen, Carcavelos, Portugal) was used following the manufacturer’s instructions. Briefly, spongy-like hydrogels were incubated with 0.5 mL of ultra-pure water for 1 h at 37 °C and then frozen at −80 °C for 24 h. Afterward, materials were heated to 70 °C for 30 min, dissolved with the help of a vortex and centrifuged for 5 min at 1000× *g*. The supernatant with the DNA content was collected. Each well of a white 96-well plate received 28.7 μL of each cell lysate followed by the addition of 100 μL of 1× Tris-EDTA buffer and 78.3 μL of PicoGreen reagent. After a 10 min incubation at RT, fluorescence was read at 480/520 nm using a Synergy HT plate reader (BioTek, Winooski, VT, USA). The concentration of DNA present in each sample was determined against a standard curve. The following equation (Equation (2)) was used to quantify the entrapment efficiency:

Entrapment efficiency (%) = DNA_f_/DNA_i_ × 100 (2)

### 2.11. Cell Survival and Cytoskeleton Organization Analysis

After three and seven days, cell-laden spongy-like hydrogels were incubated with calcein-AM (Ca-AM, 1 μg/mL, Invitrogen, Carcavelos, Portugal) and propidium iodide (PI, 2 μg/mL, Invitrogen, Carcavelos, Portugal) for 1 h at 37 °C in a humidified tissue culture incubator with 5% CO_2_ atmosphere. Then, constructs were fixed and counterstained with DAPI (0.02 mg/mL) and the percentage of live/dead cells was assessed with a Leica TCS SP8 confocal microscope. The number of live and dead cells for each condition was quantified in five random images (three independent experiments) using the Cell Counter plugin of FIJI for ImageJ. The percentage of live cells was calculated as followed (Equation (3)):

% live cells = number of live cells/total number of cells × 100(3)

For visualization of the cytoskeleton F-actin fibers and nuclei, cells were fixed and stained with phalloidin-TRITC (0.1 mg/mL, Sigma-Aldrich, Loures, Portugal) and DAPI (0.02 mg/mL), respectively. Samples were observed with a Leica TCS SP8 confocal microscope.

### 2.12. Statistical Analysis

GraphPad Prism 8 software (La Jolla, CA, USA) was used to perform statistical analysis. Data were analyzed by Shapiro–Wilk normality test. A one-way analysis of variance (ANOVA) with a Tukey post-test was used to analyze the results with a normal distribution. Otherwise, data were analyzed with the Kruskal–Wallis test with Dunn’s multiple comparison post-test. The significance of data variability was determined with the Brown–Forsythe test. Results are presented as mean ± standard deviation (SD) and as coefficient of variation (reproducibility analysis) and the significance level between groups was set for * *p* < 0.05.

## 3. Results

### 3.1. Thermal Profile Features

The thermal profile is divided in two main phases, the cooling phase occurring until the nucleation temperature is reached, and the freezing phase which follows it, starting when the nucleation temperature is reached (Figure 2B). Thus, the cooling rate was calculated from the linear region of the cooling phase, while the freezing rate was obtained from the linear region of the profile corresponding to the beginning of the freezing slope up to the end freezing temperature (set temperature of the freezer) was reached (Figure 2B).

Thermal curves and consequently the freezing parameters (cooling rate, nucleation time and temperature, freezing rate, and end freezing time) were significantly affected by the freezing temperature (−20, −80, and −210 °C) and freezing device (Figure 2C–E) but not by the concentration of polymer used to produce the materials (Figure S1). The final freezing temperature reached was only coincident with the freezing temperature for the −20 °C condition (Figure 2C(i)). The lowest temperature reached for the −80 °C condition was −60 °C (Figure 2C(ii)). Regarding the −210 °C group (Figure 2C(iii)), the immersion time in the liquid nitrogen was not enough to reach the freezing temperature, except when the standard method was used. No measures were obtained for NFD due to the inability to properly seal the device to prevent the entrance of liquid nitrogen.

The nucleation time and the end freezing time (Figure 2D and Figure 6) successively increased in relation to the standard method for the NFD and IFD and decreased with the freezing temperature. On the other hand, the cooling rate (Figure 2E(i) and Figure 6) significantly decreased (*p* < 0.001) in relation to the standard method for the NFD and IFD. The cooling rates ranged from 0.2 to 1.5 °C/min for the −20 °C, from 0.4 to 4.4 °C/min for −80 °C, and from 4.8 to 32.2 °C/min for −210 °C. The nucleation temperature (Figure 2E(ii) and Figure 6) was affected by the freezing device but not by the freezing temperature. The nucleation temperature decreased with insulation (*p* < 0.001 for 20 °C; *p* < 0.01 for −80 °C) in relation to the standard method. The freezing rate (Figure 2E(iii) and Figure 6) followed the same trend as the cooling rate; it ranged from 0.1 to 0.8 °C/min for −20 °C, 0.2–3.6 °C/min for −80 °C, and 3.9–128 °C/min for −210 °C.

### 3.2. Effect of Freezing Conditions over the Properties of Dried Polymeric Structures and Spongy-Like Hydrogels

**Figure 3:**
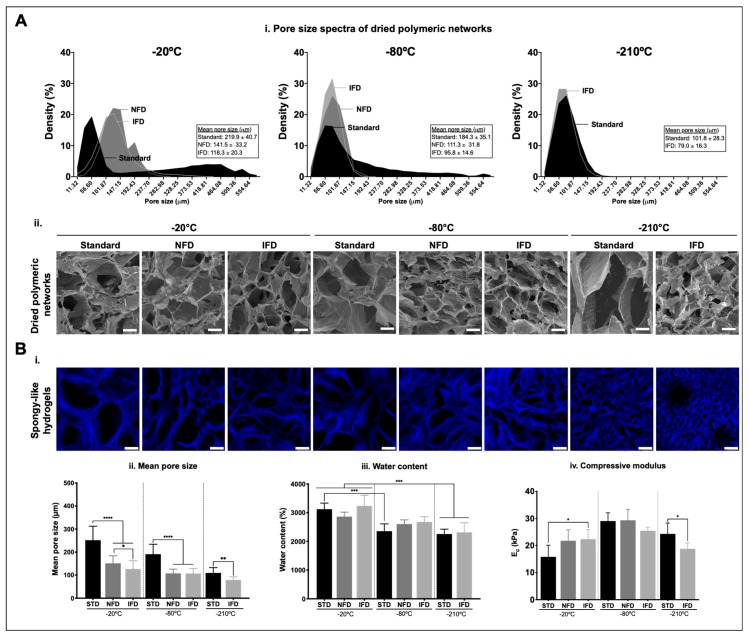
Effect of freezing conditions (standard method (STD), NFD, and IFD at −20, −80, and −210 °C) over the properties of 1.25% GG dried polymeric structures and spongy-like hydrogels. (**A**) (i) Pore size spectra and mean pore size obtained from micro-computed tomography (μ-CT) and (ii) representative scanning electron microscopy micrographs of dried polymeric structures. (**B**) (i) Representative confocal images of the microarchitecture of spongy-like hydrogels after staining with DAPI (blue). Representation of the variations of the (ii) mean pore size obtained from the analysis of images acquired by confocal microscopy, (iii) water content, and (iv) compressive modulus of spongy-like hydrogels prepared under different freezing conditions. * *p* < 0.05, ** *p* < 0.01, *** *p* < 0.001, **** *p* < 0.0001, one-way ANOVA with Bonferroni multiple comparison post-test. Scale bar = 75 µm.

The microarchitecture of GG dried polymeric networks was affected by both the freezing device and the freezing temperature (Figure 3A(i,ii) and Figure 6). Regarding the freezing device, a narrower pore size spectrum was successively observed for the materials prepared with the NFD and the IFD in relation to those prepared with the standard method. A similar tendency was observed considering the freezing temperature and independently of the GG concentrations (Figure 3A(i) and Figure S2A(i)). This impacted the mean pore size of these materials that decreased accordingly.

For the 1.25% GG dried polymeric networks (Figure 3A(i)), the pore size ranged from 11 to 577 µm with the standard method, from 11 to 306 μm with NFD, and from 11 to 441 µm with IFD for materials prepared at −20 °C. For materials prepared at −80 °C, the pore size varied from 11 to 577 µm with the standard method and from 11 to 215 μm for both NFD and IFD. The materials prepared at −210 °C, depicted pores from 11 to 238 µm with the standard method and 11 to 192 µm for IFD. Overall, similar pore sizes were attained for 0.75% GG dried polymeric structures (Figure S2A(i)) although different densities were observed for specific values. The mean pore size decreased from scaffolds prepared with the standard method to those prepared with the IFD. A similar effect was observed when the freezing temperature was diminished (Figure 3A(i,ii)) and for lower concentrations of GG (Figure S2A).

Spongy-like hydrogels were obtained from dried polymeric networks after hydration. When immersed in aqueous solutions, the dried structures took up water rapidly reaching stable values after 2 h in both α-MEM (Figure S3) and PBS (data not shown). The microstructure of the spongy-like hydrogels (Figure 3B(i)) was slightly affected after hydration but the differences in relation to the dried polymeric networks were not significant. A reduced water uptake capacity was detected for materials prepared with lower freezing temperatures (Figure 3B(iii), Figure S3 and Figure 6), around 2800%, 2500%, and 2200% of their dry weight for materials prepared at −20, −80, and −210 °C, respectively (Figure 3B(iii) and Figure 6). For 0.75% GG materials, those values changed respectively to 2500%, 2400%, and 2100%. No significant differences were detected in the mean pore size between dried polymeric networks and spongy-like hydrogels (Figure 3B(ii) and Figure 6). Again, a decrease in the mean pore size was successively observed for the materials prepared with the NFD and the IFD in relation to those prepared with the standard method. For 1.25% GG spongy-like hydrogels the mean pore size was 252, 151, and 127 µm for materials prepared at −20 °C with the standard method, the NFD and the IFD, respectively (Figure 3B(ii) and Figure 6), 191, 108, and 107 µm for those prepared at −80 °C, and 110 µm (standard) and 79 µm (IFD) when prepared at −210 °C. A similar effect was observed when the freezing temperature was diminished (Figure 3B(ii)) and for lower concentrations of GG (Figure S2B(ii) and Figure 6).

The mechanical properties of GG spongy-like hydrogels were measured after the water uptake equilibrium was reached. Both the freezing device and the freezing temperature affected the compressive modulus in a non-linear way (Figure 3B(iv), Figure S2B(iv), and Figure 6). For materials prepared with 1.25% GG, a significant (*p* < 0.05) increase and decrease in the compressive modulus was observed for materials respectively frozen at −20 and at −210 °C with the IFD in relation to the standard condition (Figure 3B(iv) and Figure 6). No significant differences in the compressive modulus were observed when materials were prepared at −80 °C. Moreover, the highest compressive modulus, ~27.7 kPa, was obtained at this temperature. The same trend was not observed for materials prepared with 0.75% GG (Figure S2B(iv) and Figure 6). While a significant decrease (*p* < 0.05) was obtained for materials frozen at −20 °C with the IFD in relation to the standard condition, no differences in the compressive modulus were observed between freezing devices, either at −80 or −210 °C (Figure S2B(iv) and Figure 6).

### 3.3. Reproducibility Analysis

**Figure 4:**
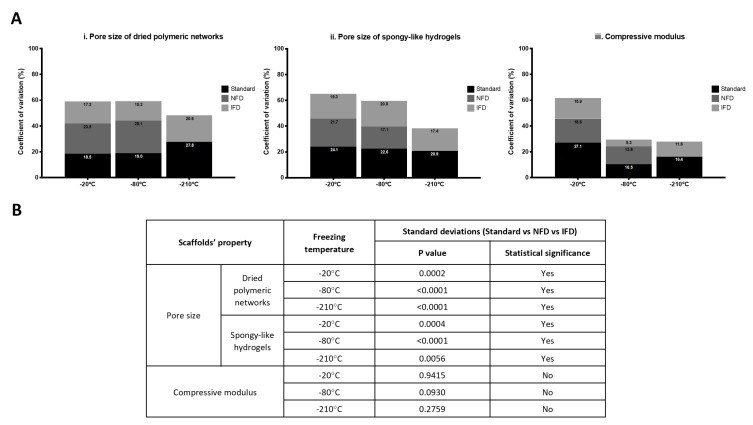
Scaffolds reproducibility. (**A**) Representation of the coefficient of variation of the pore size of 1.25% GG (i) dried polymeric networks and (ii) spongy-like hydrogels, and of the (iii) compressive modulus of spongy-like hydrogels according to the tested freezing conditions, standard method, NFD and IFD at −20, −80, and −210 °C. (**B**) *p* values obtained from the Brown–Forsythe test showing the statistical significance of the standard deviations for the pore size and compressive modulus among the different freezing devices (standard method, NFD, and IFD).

The variation of the data related to the microstructure, physicochemical, and mechanical properties of GG-based dried polymeric structures and spongy-like hydrogels was evaluated in order to assess the level of homogeneity/standardization attained using the freezing devices. There seemed to be a lower coefficient of variation for 1.25% GG materials when the freezing temperatures are lower. More importantly, a reduction in the coefficient of variation was achieved when using the freezing devices. The coefficient of variation for the pore size of the dried polymeric networks that ranged from 18.5 to 27.8 for the materials prepared with the standard method, decreased to 15.2–20.6 when IFD was used (Figure 4A(i)). Similarly, it decreased from 20.9–24.1 to 17.4–20.0 for the spongy-like hydrogels (Figure 4A(ii)). Notwithstanding, the ranges of the coefficient of variation of the compressive modulus were 15.5–27.1 and 15.9–5.3, respectively for the standard method and IFD condition (Figure 4A(iii)). The analysis of the standard deviations (Figure 4B) confirmed significant differences in the pore size variation of both dried polymeric networks and spongy-like hydrogels produced with the different freezing systems and independently of the freezing temperature. The same tendency was observed for all the properties of 0.75% GG materials (Figure S4).

### 3.4. hDFbs Survival and Viability within Spongy-Like Hydrogels

**Figure 5:**
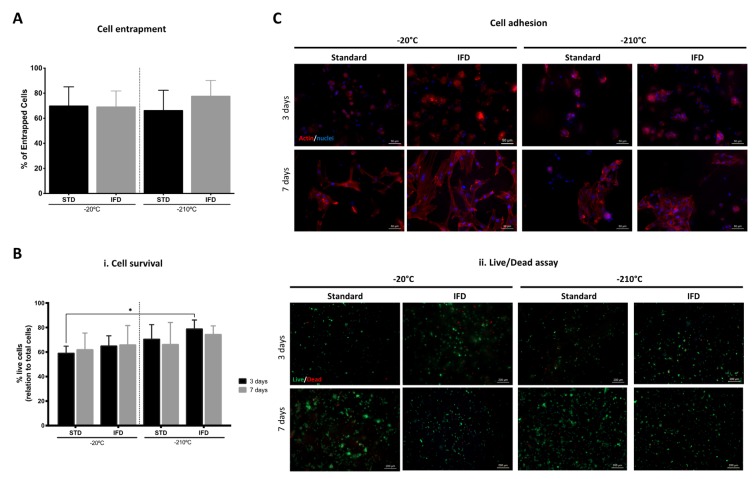
Effect of freezing conditions (standard method (STD), NFD, and IFD at −20 and −210 °C) over human dermal fibroblasts’ (hDFbs) behavior in 1.25% GG spongy-like hydrogels. (**A**) Representation of the entrapment efficiency 24 h after cell seeding. (**B**) (i) Representation of the percentage of the live cells three and seven days of culture. (ii) Representative fluorescence microscopy images showing the dead (red) and the live (green) cells, respectively stained with propidium iodide (PI) and calcein-AM (Ca-AM) after three and seven days of culture. Scale bar = 200 µm. (**C**) Representative fluorescence microscopy images of hDFbs after three and seven days of culture showing the F-actin cytoskeleton (phalloidin-TRITC, red) and nuclei (DAPI, blue). Scale bar = 50 µm. Data were obtained from three independent experiments with three replicates for each condition, * *p* < 0.05, two-way ANOVA and Bonferroni’s post-hoc test.

Regarding the capacity of spongy-like hydrogels to support hDFB entrapment, adhesion, and viability, after 24 h of cell seeding, 70% of the total number of seeded cells remained in the structure (Figure 5A). No differences were observed when cells were entrapped on materials prepared with different freezing devices and different freezing temperatures (Figure 5A). After three days of culture, the percentage of live cells was higher in the materials prepared in the IFD at the lowest temperature, but a significant difference (*p* < 0.05) was only observed between the materials frozen at −20 °C using the standard method and those frozen at −210 °C in the IFD (Figure 5B(i)). Overall, the percentage of live cells was higher than 60% in all conditions after three and seven days (Figure 5B). Regarding cell morphology (Figure 5C), cells showed a round-like shape after three days but a well-organized actin cytoskeleton and a spread and spindle-like morphology after seven days in all conditions. Moreover, hDFbs seemed clustered after three days but after seven days, cells were able to colonize the spongy-like hydrogels, organizing in higher-density adherent colonies in the materials prepared at −210 °C (Figure 5C). The same tendency was observed for 0.75% GG spongy-like hydrogels (Figure S5).

### 3.5. Mapping of Thermal Parameters Used for Scaffold Preparation and Scaffolds’ Properties

**Figure 6:**
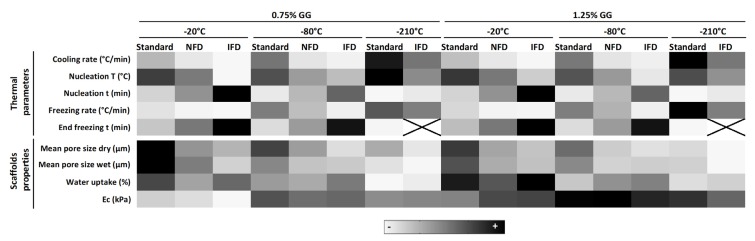
Heat map showing the relative effect of the freezing devices and temperatures, and polymer concentration on the different thermal parameters and scaffolds’ properties. T = temperature, t = time.

A gradient color mapping of thermal parameters used for scaffold preparation and scaffolds’ properties is illustrated in Figure 6. Considering the different freezing devices (standard/NFD/IFD), the cooling rate, nucleation temperature, and freezing rate decrease from the standard to the IFD. In opposition, the nucleation time and end freezing time increased (standard < NFD < IFD). These variations impacted scaffolds’ properties by decreasing the mean pore size in dry and wet state (standard < NFD < IFD) but did not significantly affect the water uptake. These profiles are valid for any of the temperatures tested as well as for both 1.25% and 0.75% GG concentrations. Interestingly, there seemed to be a tendency for the modulus to be less affected by the freezing device when lower temperatures were used.

Within each freezing device, an increase of the cooling rate, nucleation temperature, and freezing rate was observed as the freezing temperature decreases (−20 °C < −80 °C < −210 °C) while the opposite was observed for the nucleation time and end freezing time (−20 °C > −80 °C > −210 °C). These impacted the mean pore size of scaffolds in the dry and wet state, and the water uptake, all of them reduced for lower freezing temperatures (−20 °C > −80 °C > −210 °C). In what concerns the modulus, there seemed to be a tendency to be less affected when lower temperatures were used, however, higher amounts of GG led to an increase in this mechanical property.

## 4. Discussion

In the last years, we have been assessing the potential of GG spongy-like hydrogels for a range of tissue engineering and regenerative medicine applications [15,16,17,18,19,20,21,22,23]. Although the whole procedure for preparing spongy-like hydrogels is well defined, it is important to recognize that batch-to-batch variability affects the final scaffolds’ architecture and overall properties. Thus, we hypothesized that these variations are dependent on the freezing conditions. The freezing stage is the most critical step during spongy-like hydrogels’ formation since it determines ice nucleation and crystal growth, thus influencing their particular micro-architecture. As the GG dried polymeric structures are formed from precursor hydrogels, the water content and the way ice crystals are formed during freezing and then sublimed with the freeze-drying determine pore size and overall porosity [15]. Ice crystals start to form from a nucleation point, and then grow and merge until a solid–liquid system equilibrium is reached [31]. Ice crystal nucleation and growth is highly influenced by the freezing thermodynamics [31]. Therefore, freezing parameters, such as nucleation and freezing time, cooling and freezing rate, and nucleation temperature play a key role.

Aiming to understand those freezing parameters and consequentially to be able to tailor dried polymeric network/spongy-like hydrogels’ properties, different freezing devices/strategies were considered. In addition to the standard approach that has been followed to prepare those structures [32], a non-insulated and highly thermo-conductive freezing device (NFD) and an insulated freezing device (IFD) were used at −20, −80, and −210 °C. The NFD and the IFD were designed considering thermo-conductive metals (aluminum and copper) to allow the control of heat transference in a more homogeneous way. Thus, hydrogels were placed in a 24-well (comparable to the polystyrene one used in the standard method) aluminum plate, which was then held between two (top/bottom) copper plates. In the case of IFD, an extra insulating layer (lid/base) was added to the system. This means that, despite the use of the thermo-conductive materials, a higher thermal resistance was also added. As the temperature difference is the driving force for heat transfer, the larger the difference, the higher is the transfer rate and the faster is the freezing [33]. Thus, higher energy was required for heat transfer with the addition of thermal plates and insulation materials (standard < NFD < IFD). As a consequence, both cooling and freezing rate decreased (standard > NFD > IFD), thus also impacting the nucleation and end freezing time. The time for latent heat removal increased and, thus, the nucleation time increased (standard < NFD < IFD) and the nucleation temperature decreased. Consequently, the time for crystal growth (after end freezing time) decreased, which culminates in a reduced pore size after freeze-drying. Moreover, the thermal hold for materials prepared with the standard method was higher, since hydrogels reached the end freezing temperature faster. Higher thermal holds impact ice growth, as they create larger ice crystals at the expense of smaller ones [34,35]. Therefore, a broader pore size range and higher mean pore size were obtained when the time for ice growth was higher (standard method), and the narrowest pore size spectrum and the lower mean pore size (IFD) occurred when the time for ice growth was lower. With the decrease of the cooling and freezing rates due to the use of the freezing devices, a more controlled freezing was achieved, as reflected by the smaller coefficients of variation as well as statistical significances of the standard deviations. As expected, polymer concentration did not affect the assessed freezing parameters, but affected the properties of the scaffolds, particularly the pore size range and the compressive modulus. This is also in agreement with previous works that did not show significant differences in the cooling rate with the increase of polymer concentration but showed a reduced pore size due to a lower nucleation temperature [36]. These same variations observed in the mean pore size for the dried polymeric networks were also noticed on the wet structures, as the mean pore size decreased for the IFD in relation to the standard method. Moreover, when comparing the mean pore size of the dried polymeric networks and the spongy-like hydrogels, a slight increase was observed. This effect that resulted from the water uptake of the pore walls of the dried scaffolds was not statistically significant. Moreover, these differences in the pore size obtained from the different freezing methods did not affect the capacity of hDFbs to survive, adhere, and colonize within these structures, as already shown by others [15]. The hDFbs adhered to the pore walls and organized their cytoskeleton, exhibiting their typical morphology along the time of culture.

When comparing different freezing temperatures within each device/method, lower temperatures were associated with larger intervals between the settled and the hydrogel temperatures. These differences were responsible for higher cooling and freezing rates, leading to a faster nucleation and to less time to stabilize and reach the final temperature. Cooling rates have been directly linked to the isotropy/anisotropy of scaffolds. Dried polymeric structures obtained with −20 and −80 °C freezing procedures depicted an open honeycomb-like structure, whereas a parallel laminar porous structure was obtained at −210 °C. Nonetheless, this was only observed for samples prepared by the standard method associated with the highest cooling rate. This is in concordance with other works that showed that rapid cooling rates at very low temperatures, such as liquid nitrogen, cause reduction of the pore size and orientation of the ice crystals [26,37]. With regard to the nucleation temperature, no differences were observed when directly comparing the same device/method at different freezing temperatures. These results are in line with others that also did not show differences in the nucleation temperature when collagen freezing was varied from −20 to −40 °C [25]. Interestingly, although nucleation temperature is often cited as a predictive measure of pore size [38], the results herein presented, as also shown by others [25], confirm that, in opposition to nucleation temperature, the pore size of the obtained scaffolds decreased for lower freezing temperature. This can be explained by the efficiency of latent heat removal (dependent on the freezing temperature) that determines the final pore size due to the decreased time of ice nucleation and growth [39].

Herein, we demonstrated the link between the thermal parameters and the properties of the dried polymeric networks/spongy-like hydrogels by showing that altering the freezing system and the freezing temperature changed the microstructure of the GG structures. This is particularly evident for the pore size, which is justified by the expected differences in the efficiency of latent heat removal from the hydrogel [25]. From the performed analysis we could confirm that by changing either the method (standard vs. IFD) or the freezing temperature (from −20 to −210 °C within the same method) spongy-like hydrogels with lower pore size can be obtained. While changing the method (standard vs. IFD) did not affect water uptake capability, when the freezing temperature is varied from −20 to −210 °C (within the same method), lower pore size is accompanied with lower water content. Interestingly those variations did not significantly affect the compressive modulus of the spongy-like hydrogels.

The mapping of the effect of the freezing device and temperatures will allow guiding the preparation of spongy-like hydrogels with a specific pore size knowing whether and how the water content is affected without compromising the compressive modulus. Overall, the freezing devices did not disturb the characteristic features of the spongy-like hydrogels but allowed obtaining highly reproducible materials and a powerful procedure to fine tune their microarchitecture according to specific needs.

## Supplementary Materials

The following are available online at https://www.mdpi.com/2073-4360/12/2/329/s1, Figure S1: Freezing profiles of 0.75% GG hydrogels, Figure S2: Effect of freezing conditions (standard method, NFD, and IFD at −20, −80, and −210 °C) over the properties of 0.75% GG dried polymeric structures and spongy-like hydrogels., Figure S3: Water uptake of 1.25% GG spongy-like hydrogels at −20, −80, and −210 °C using different freezing systems, Figure S4: Scaffolds reproducibility, Figure S5: Effect of freezing conditions (standard method, NFD, and IFD at −20, −80, and −210 °C) over hDFb behavior in 0.75% GG spongy-like hydrogels.
