# Supplementary material for: Tailoring Gellan Gum Spongy-Like Hydrogels’ Microstructure by Controlling Freezing Parameters
Source: Polymers (Basel). 2020 Feb 5;12(2):329. doi: 10.3390/polym12020329 (PMC7077413; doi:10.3390/polym12020329)
Supplement: Supplementary file 1 [file polymers-12-00329-s001.pdf]

polymers

MDPI

Article

Tailoring gellan gum spongy-like hydrogels' microstructure by controlling freezing parameters

Helena R. Moreira[1,2,3], Lucília P. da Silva[1,2], Rui L. Reis[1,2,3] and Alexandra P. Marques[1,2,3,] *

[1] 3B's Research Group, I3Bs – Research Institute on Biomaterials, Biodegradables and Biomimetics, University of Minho, Headquarters of the European Institute of Excellence on Tissue Engineering and Regenerative Medicine; helena.moreira@i3bs.uminho.pt (H.R.M); lucilia.silva@i3bs.uminho.pt (L.P.d.S.); rgreis@i3bs.uminho.pt (R.L.R.); apmarques@i3bs.uminho.pt (A.P.M.)
[2] ICVS/3B's – PT Government Associate Laboratory, Braga/Guimarães 4805-017, Portugal;
[3] The Discoveries Centre for Regenerative and Precision Medicine, Headquarters at University of Minho, Avepark 4805-017 Barco, Guimarães, Portugal
* Correspondence: apmarques@i3bs.uminho.pt; Tel.: (+351252510900)

Supplementary figures

A

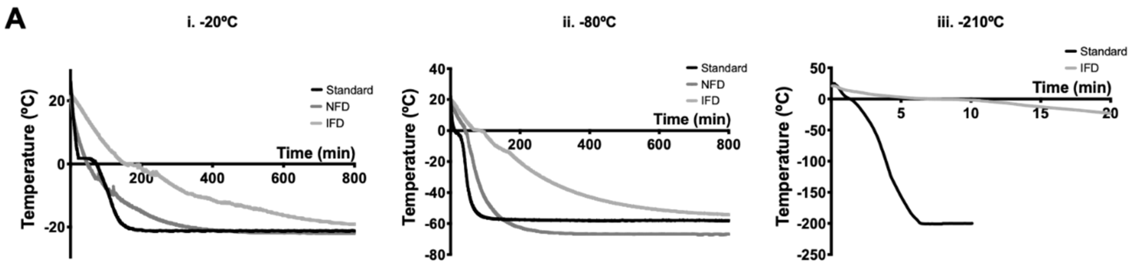

B

Freezing temperature (°C)	Freezing device	Nucleation time (min)	End freezing time (min)
-20	Standard	25	190
	NFD	70	410
	IFD	165	780
-80	Standard	10	90
	NFD	44	300
	IFD	100	720
-210	Standard	1	3
	IFD	10	-

C

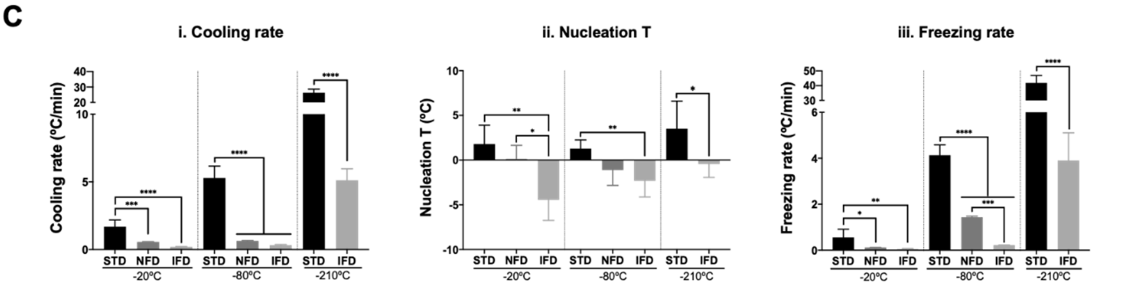

Figure S1. Freezing profiles of 0.75% GG hydrogels. **(A)** Mean temperature profiles of GG hydrogels during freezing at (i) -20°C, (ii) -80°C and (iii) -210°C with the 3 different freezing molds. **(B)** Characteristic values of the freezing of GG hydrogels. **(C)** Effect of both temperature and freezing mold on the (i) Cooling rate, (ii) Nucleation T and (iii) Freezing rate of GG hydrogels. Data was

obtained from 7 different measurements, * p < 0.05, ** p < 0.01, *** p < 0.001, **** p < 0.0001, One-way ANOVA with Bonferroni multiple comparison post-test.

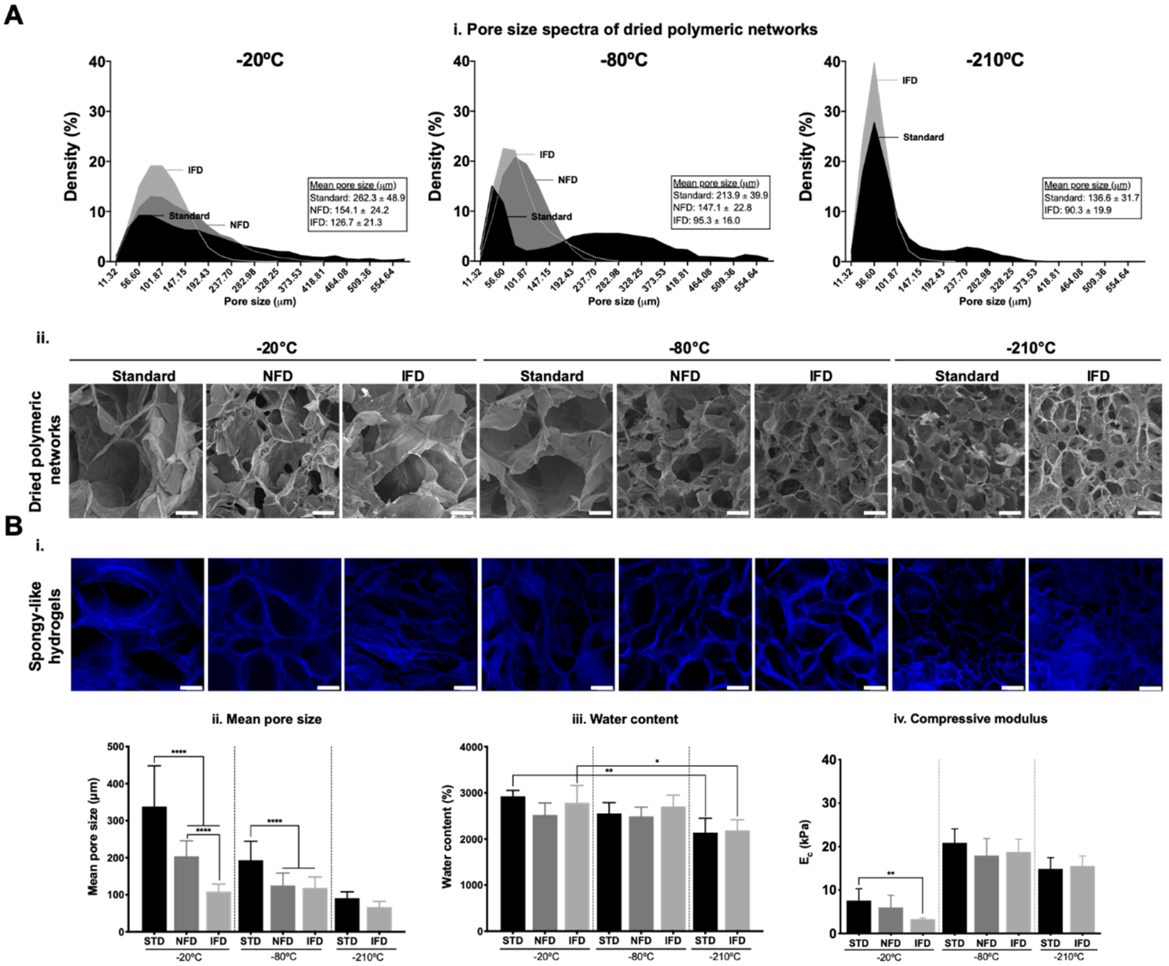

Figure S2. Effect of freezing conditions (standard method, NFD and IFD at -20°C, -80°C and -210°C) over the properties of 0.75% GG dried polymeric structures and spongy-like hydrogels. **(A)** (i) pore size spectra and mean pore size obtained from *μ-CT* and (ii) representative scanning electron microscopy micrographs of dried polymeric structures. **(B)** (i) Representative confocal images of the microarchitecture of spongy-like hydrogels after staining with DAPI (blue). Representation of the variations of the (i) mean pore size analyzed from images obtained by confocal microscopy, (ii) water content and (iii) compressive modulus of spongy-like hydrogels prepared under different freezing conditions. * p < 0.05, ** p < 0.01, *** p < 0.001, **** p < 0.0001, One-way ANOVA with Bonferroni multiple comparison post-test. Scale bar = 75 μm.

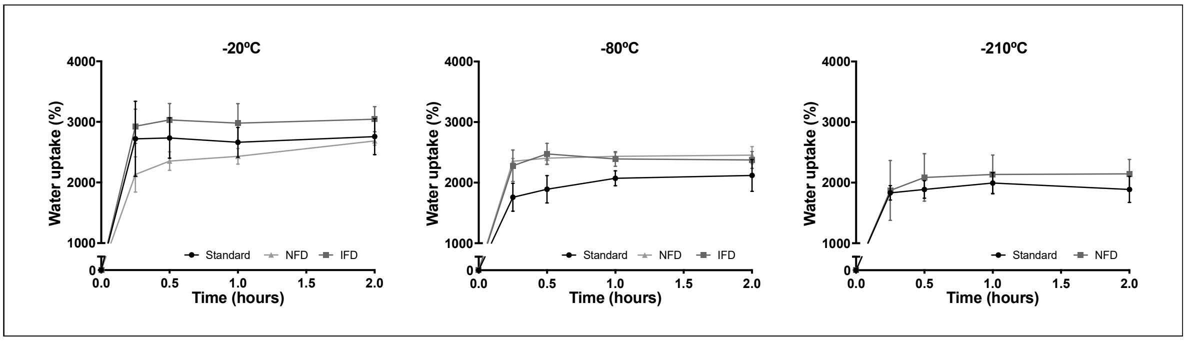

Figure S3. Water uptake of 1.25% GG dried polymeric structures prepared at -20°C, -80°C and -210°C and using different freezing systems.

A

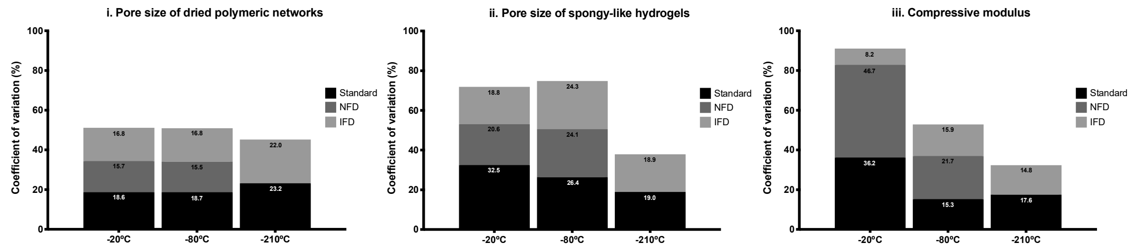

B

Scaffolds' property		Freezing temperature	Standard deviations (Standard vs NFD vs IFD)	
			P value	Statistical significance
Pore size	Dried polymeric networks	-20°C	<0.0001	Yes
		-80°C	<0.0001	Yes
		-210°C	0.0062	Yes
	Spongy-like hydrogels	-20°C	<0.0001	Yes
		-80°C	<0.0001	Yes
		-210°C	0.1309	No
Compressive modulus		-20°C	0.1279	No
		-80°C	0.7800	No
		-210°C	0.8126	No

Figure S4. Scaffolds reproducibility. **(A)** Representation of the coefficient of variation of the pore size of 0.75% GG (i) dried polymeric networks and (ii) spongy-like hydrogels, and of the (iii) compressive modulus of spongy-like hydrogels according to the tested freezing conditions, standard method, NFD and IFD at -20°C, -80°C and -210°C. **(B)** *p* values obtained from the Brown-Forsythe test showing the statistical significance of the standard deviations for the pore size and compressive modulus among the different freezing devices (standard method, NFD and IFD).

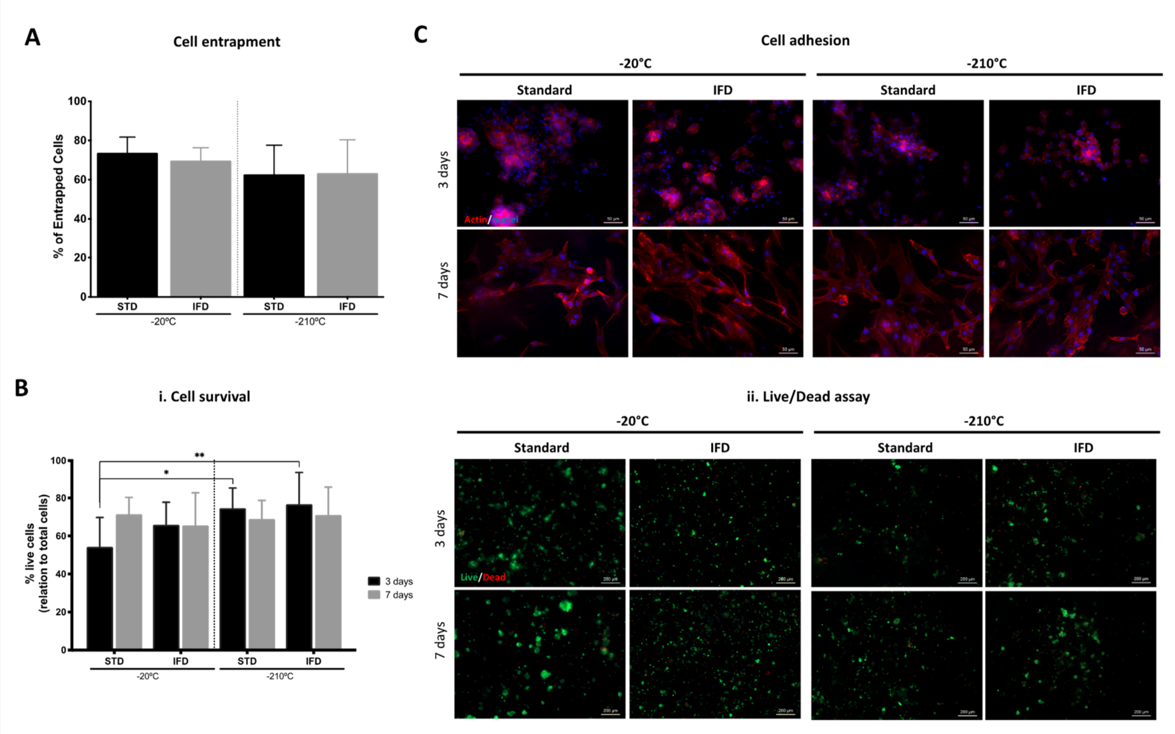

Figure S5. Effect of freezing conditions (standard method, NFD and IFD at -20°C and -210°C) over hDFbs behaviour in 0.75% GG spongy-like hydrogels. **(A)** Representation of the entrapment efficiency 24 hours after cell seeding. **(B)** (i) Representation of the percentage of the live cells 3 and 7 days of culture. (ii) Representative fluorescence microscopy images showing the dead (red) and the live

(green) cells, respectively stained with PI and Ca-AM after 3 and 7 days of culture. Scale bar = 200 μm. **(C)** Representative fluorescence microscopy images of hDFBs after 3 and 7 days of culture showing the F-actin cytoskeleton (phalloidin-TRITC, red) and nuclei (DAPI, blue). Scale bar = 50 μm. Data was obtained from three independent experiments with three replicates for each condition, * $p < 0.05$, two-way ANOVA and Bonferroni's post-hoc test.
